# Spontaneous Nosocomial* Pseudomonas aeruginosa* Meningitis Presenting as Trismus

**DOI:** 10.1155/2017/8705860

**Published:** 2017-05-08

**Authors:** C. J. Parr, J. Wheeler, A. Sharma, C. Smith

**Affiliations:** ^1^Department of Internal Medicine, Max Rady College of Medicine, University of Manitoba, Winnipeg, MB, Canada; ^2^Section of General Internal Medicine, Max Rady College of Medicine, University of Manitoba, Winnipeg, MB, Canada; ^3^Section of Infectious Diseases, Max Rady College of Medicine, University of Manitoba, Winnipeg, MB, Canada

## Abstract

We describe the case of a 78-year-old female receiving adjuvant postsurgical chemotherapy for colon adenocarcinoma who spontaneously developed nosocomial* Pseudomonas* meningitis causing severe trismus. The patient was initially admitted for ileus, developing neck stiffness and trismus on the thirteenth day of admission. Cerebrospinal fluid grew pansensitive* Pseudomonas aeruginosa*. Magnetic resonance imaging of the brain was consistent with bilateral subacute infarcts secondary to meningitis. The patient responded well to 21 days of broad spectrum antimicrobial therapy modified to ceftazidime alone following speciation and sensitivity. Outpatient follow-up at 46 days revealed normal maximal mouth opening with the ability to chew and tolerate a full diet. Trismus is a motor disturbance of the trigeminal nerve with difficulty in opening the mouth. Infectious etiologies commonly described include tetanus, odontogenic infections, or deep neck space infections. This is the first reported case of simultaneous nosocomial* Pseudomonas* meningitis and trismus in a patient with no history of neurosurgery or lumbar spinal manipulation.

## 1. Introduction

Trismus has been described as a motor disturbance of the trigeminal nerve with restricted maximal mouth opening (MMO), characteristic of tetanus [1]. In the most recent literature, the term trismus has been applied generally to mean any condition in which mouth opening is inadequate [2]. While typical MMO range is between 40 and 60 mm, it is sex and age dependent. Nevertheless, the consensus is that any interincisor measurement below 34 mm is clinically abnormal [3]. Trismus may present as functional disability with impairments in yawning, eating, or speaking. Limited mouth opening may even be life-threatening with diminished clearing of oral secretions or in the event of required emergent intubation [4].

The etiology of trismus is broad and includes both intra-articular and extra-articular conditions. Causes include infection, trauma, dental treatment, temporomandibular joint disorder, neoplasia, CNS lesions, radiation, and drugs. Infectious causes are considered extra-articular and commonly include tetanus, odontogenic infections, or deep neck space infections.

There are few reports in the literature of trismus in the setting of meningitis [5, 6]. As far as we know, there are no published cases of trismus secondary to nosocomial meningitis (greater than 48 hours after admission or within one week of discharge [7, 8]). In this report, we present the case of an elderly female without neurosurgical or lumbar spinal manipulation who developed* Pseudomonas aeruginosa* meningitis while in hospital, leading to trismus.

## 2. Case Presentation

A 78-year-old female had initially presented to hospital with progressive nausea, vomiting, and decreased oral intake. A computed tomography (CT) scan of the abdomen and pelvis showed diffuse ileus. She was admitted to the acute surgical care service and treated conservatively with nasogastric tube suction, antiemetics, and intravenous fluids.

**Figure 1:**
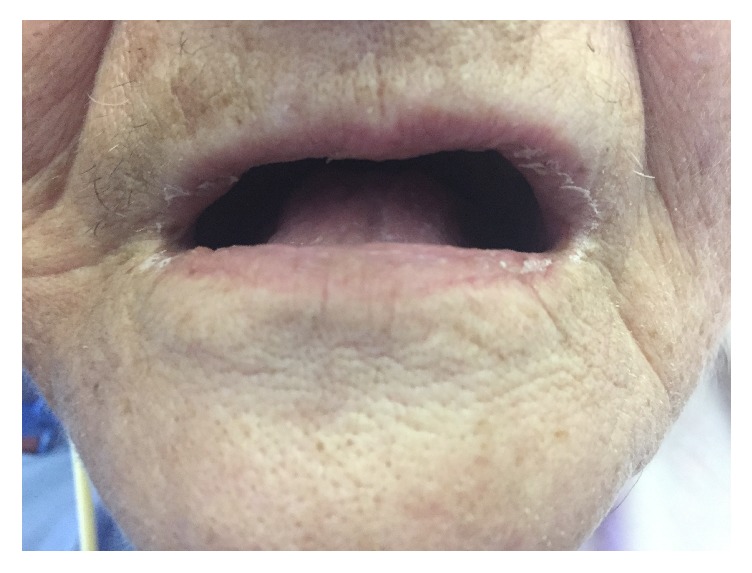
Maximum lip-to-lip mouth opening size of 12 mm on admission day 28. The patient was initially unable to speak or chew.

The patient had been improving clinically with resolution of ileus until admission day 13, when she acutely developed fever and altered level of consciousness. CT head and angiography were unremarkable. White blood cell count was 11.8 × 10^9^/L with neutrophilic predominance. Blood cultures were negative. The patient was started on intravenous (IV) ceftriaxone (2 g every 24 hours) for presumed urosepsis secondary to* Escherichia coli* and* Klebsiella pneumoniae*. Internal Medicine was consulted on admission day 15 for ongoing fever and delirium. The patient was febrile, inconsistently following commands, with nuchal rigidity and a positive Kernig's sign. Fundi were poorly visualized on nondilated fundoscopy. With passive opening of the patient's mouth, MMO was less than 20 mm with bilateral firm and spastic masseters, resulting in an inability to speak and difficulty clearing secretions (Figure 1). It was impossible to forcefully open her mouth as the muscles of mastication were contracting involuntarily. CT of the neck ruled out anatomical cause for trismus. Endoscopic evaluation showed bilateral vocal cord motion with patent subglottis and upper trachea without masses or purulence. A lumbar puncture was performed to rule out meningitis or encephalitis. Cerebrospinal fluid (CSF) was turbid, with total nucleated cell count of 9900 with neutrophilic predominance, protein 1.94 g/L, and gram stain 4+ polymorphonuclear leukocytes.* Clostridium tetani* toxin antibody was 0.04 IU/mL, indicating uncertain immunization protection.

The patient had recently been diagnosed with colon adenocarcinoma. Right hemicolectomy was performed three months earlier and adjuvant capecitabine had been initiated two months ago but was held due to significant nausea three weeks prior to her admission. She had no prior history of penetrating head trauma, neurosurgery, otorhinologic surgery, lumbar spinal puncture, odontogenic infection, or intravenous drug use. The patient had not recently been exposed to strychnine, dopamine antagonists, or anticholinergic drugs. The patient denied any history of a prior wound or injury that could suggest a portal of entry for* C. tetani*. Notably, she had not been vaccinated for tetanus in the last 10 years.

Empiric metronidazole (500 mg IV every 8 hours), ampicillin (2 g IV every 6 hours), and vancomycin (750 mg IV every 12 hours) were added to initial ceftriaxone monotherapy. After consultation with Infectious Diseases, antimicrobial therapy was modified to meropenem (500 mg IV every 6 hours), vancomycin (750 mg IV every 12 hours), ampicillin (2 g IV every 6 hours), and acyclovir (550 mg IV every 8 hours) pending blood and CSF culture results. The CSF culture was positive for the detection of gram-negative bacilli after 19 hours of incubation in a BacT/ALERT PF Plus Culture Bottle (bioMérieux, St. Laurent, QC, Canada). The broth was subcultured to sheep blood agar, chocolate agar, and* Brucella* agar with vitamin K. After 5 hours of incubation, the early bacterial growth was identified as* Pseudomonas aeruginosa* by matrix assisted laser desorption/ionization time-of-flight mass spectrometry (MALDI-TOF) using the Bruker Biotyper (Bruker Daltonics, Bremen, Germany). After 18 hours of incubation, the culture was confirmed as monomicrobial and repeated identification using MALDI-TOF confirmed the identification of* P. aeruginosa*. Antimicrobial susceptibility testing was performed using Vitek n208 antimicrobial susceptibility card (bioMérieux, St. Laurent, QC, Canada). The isolate was susceptible to ceftazidime, ciprofloxacin, gentamicin, piperacillin-tazobactam, tobramycin, and meropenem. Once the antimicrobial susceptibility results became available, the antimicrobials were modified to ceftazidime (2 g IV every 8 hours) monotherapy for a total duration of 21 days.

**Figure 2:**
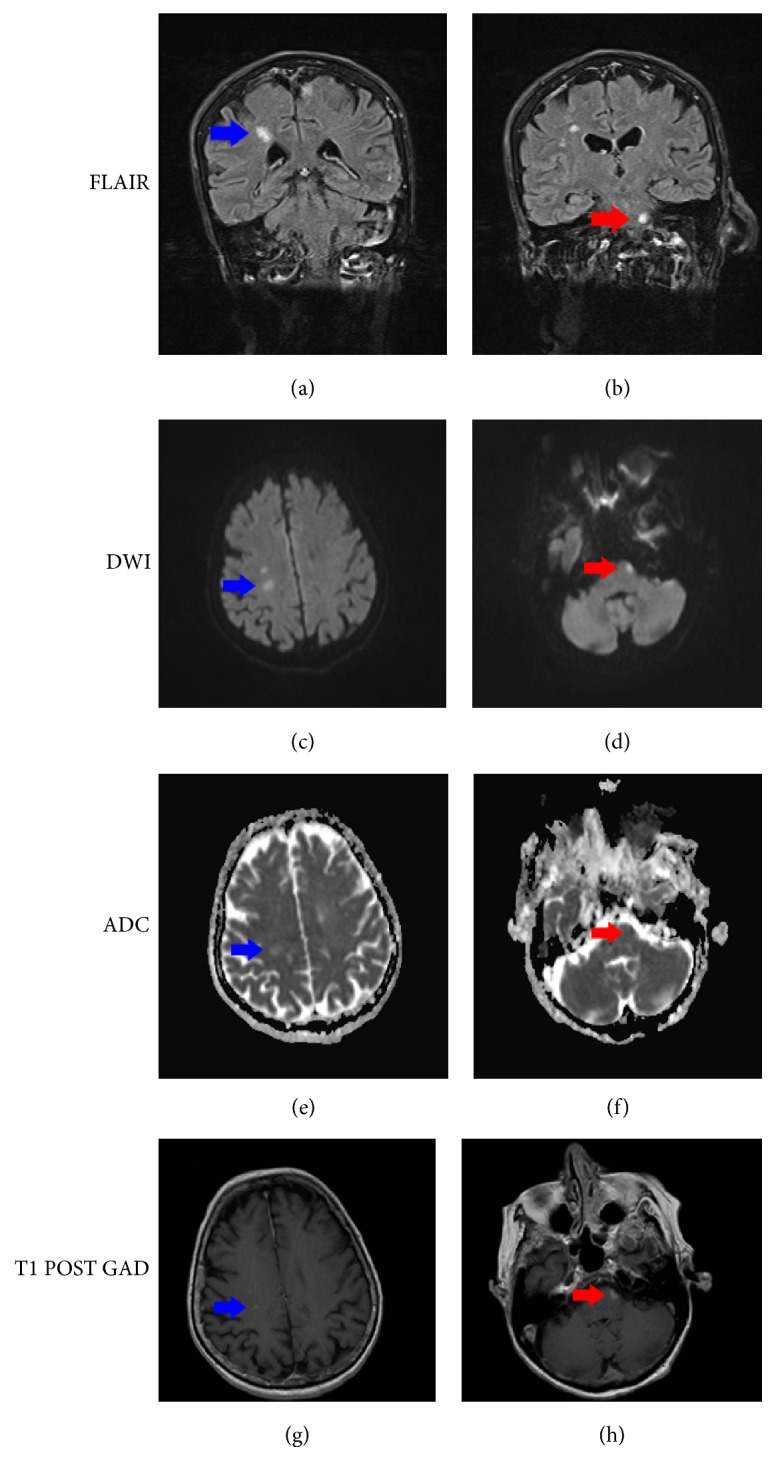
Representative MRI images showing infarcts in the right parietal lobe (blue arrow; images (a), (c), (e), and (g)) and left ventral pons (red arrow; images (b), (d), (f), and (h)). The right parietal lesion shows DWI signal change without corresponding ADC changes, consistent with subacute infarct. The left pontine lesion shows both DWI and ADC signal changes suggesting acute infarct. FLAIR, fluid-attenuated inversion recovery. DWI, diffusion weighted imaging. ADC, apparent diffusion coefficient. T1 POST GAD, T1 postgadolinium.

The patient's delirium and nuchal rigidity resolved rapidly but trismus persisted. Panoramic radiographs of her upper and lower teeth were unremarkable. Clinical evaluation by dentistry did not reveal an odontogenic etiology. Gadolinium infused magnetic resonance imaging (MRI) of the spine and brain showed increased signal attenuation in the central pons, left ventral pons, and white matter of the frontal and parietal lobes bilaterally in addition to leptomeningeal enhancement (Figure 2). This was most in keeping with acute pontine and subacute parietal infarcts possibly related to meningitis or vasculitic injury. It is unlikely that these lesions were brain abscesses because there were no ring-enhanced lesions, no tissue edema, and variable gadolinium enhancement in the contrast-enhanced T1-weighted images. There was no evidence of epidural abscess, diskitis, or osteomyelitis.

With warm compresses and jaw physiotherapy, the patient regained the ability to speak and chew and was discharged with a MMO of 26 mm. Follow-up appointment 46 days after discharge revealed a MMO in the normal range. The patient was able to tolerate her previous diet and no longer appeared cachectic.

## 3. Discussion

The described case is unique and has several interesting characteristics. The simultaneous development of trismus and meningitis several weeks after admission to hospital for an unrelated condition was unexpected. Consideration was initially given to tetanus, given the uncertain immunization history. Nevertheless, there was no obvious portal of entry, characteristic generalized muscle spasms and abdominal rigidity were absent, and the patient's* C. tetani* antibody was above the generally accepted protective concentration of 0.01 IU/mL [9–12]. It should be noted, however, that several clinicians have proposed that the protective antibody titer level be revised, with no current consensus [11, 13]. With a CSF sample that had marked neutrophilic pleocytosis and growing* P. aeruginosa*, it became evident that meningitis was a likely contributor.

Nosocomial meningitis has been defined as developing 48 hours after admission or within one week of discharge [8, 14]. It is typically seen as a complication of craniotomy, placement of internal or external ventricular catheters, otorhinologic surgery, lumbar puncture, or head trauma [15]. It can seldom present as hematogenous spread from a distant focus of infection or secondary from local sources such as mastoiditis. Remarkably, our patient did not have any history of neurosurgical instrumentation. Despite the patient being treated for* Klebsiella* bacteriuria, there was no evidence of a distant focus of* Pseudomonas* infection. This was confirmed with extensive evaluation including an MRI not showing any evidence of epidural abscess, diskitis, or osteomyelitis. Likewise, with investigations including CT of the brain, facial bones and neck, oropharyngeal endoscopy, panoramic dental radiographs, and dental examination, there was no obvious local source. Given that* Pseudomonas* is the most common pathogen in nosocomial spontaneous gram-negative bacillary meningitis [8], it is imperative that all patients who develop nosocomial meningitis be initially treated with antibiotics to empirically cover* Pseudomonas* until pathogen identification and antimicrobial susceptibilities become available.

Cerebral infarction is not an uncommon complication of meningitis. The trigeminal motor nucleus arises from the pons and the motor component runs along the mandibular division of the trigeminal nerve. Patients with known focal central nervous system lesions of the trigeminal nerve system in the medulla and pons may have dissynergism of the masticatory muscles on the basis of clinical and EMG findings, leading to trismus [2, 16]. In our case, the MRI of the brain revealed acute infarcts in the central and left pons in addition to the bilateral frontal white matter lesions. Hence, we hypothesize that our patient developed neurogenic trismus secondary to meningitis-induced infarction of the pons.

Existing case reports of trismus in the setting of meningitis have been reported in the setting of chronic tuberculous meningitis [5] and with iatrogenic meningitis from recent lumbar instrumentation [6]. In this case study, we have emphasized the need to consider meningitis as a cause of cranial nerve dysfunction including trigeminal nerve injury causing trismus. Additionally, it is critically important to empirically treat hospital acquired meningitis with antibiotics that cover gram-negative bacillary pathogens including* Pseudomonas*.
